# The reliability and validity of the Japanese version of the Daily Record of Severity of Problems (J-DRSP) and Development of a Short-Form version (J-DRSP (SF)) to assess symptoms of premenstrual syndrome among Japanese women

**DOI:** 10.1186/s13030-021-00208-z

**Published:** 2021-03-18

**Authors:** Yumie Ikeda, Miho Egawa, Kazuya Okamoto, Masaki Mandai, Yoshimitsu Takahashi, Takeo Nakayama

**Affiliations:** 1 Department of Health Informatics, Kyoto University School of Public Health, Konoecho Yoshida, Sakyo-ku, Kyoto City, 606-8303 Japan; 2 Department of Obstetrics and Gynecology, Kyoto University Hospital, 54 Kawaramachi Shogoin, Sakyo-ku, Kyoto City, 606-8397 Japan; 3 Department of Medical Informatics, Kyoto University Hospital, 54 Kawaramachi Shogoin, Sakyo-ku, Kyoto City, 606-8397 Japan

**Keywords:** Premenstrual syndrome, Japanese, DRSP, Reliability, Validity

## Abstract

**Purpose:**

To assess the validity and reliability of the Japanese version of the Daily Record of Severity of Problems (J-DRSP, 24 items) for evaluating symptoms of premenstrual syndrome (PMS), and to develop a short form version of the J-DRSP.

**Methods:**

Using the “DRSP-JAPAN” smartphone app, we collected daily J-DRSP records from cycle day − 6 (CD − 6) to CD 10, with CD 1 representing the menstruation onset date. Factorial validity (exploratory factor analysis: EFA, confirmatory factor analysis: CFA) and criterion validity were examined, and test-retest reliability (intraclass correlation: ICC) evaluated. The short-form version of the J-DRSP was developed using classical test theory.

**Results:**

In total, 304 women participated and 243 recorded symptoms on at least 4 days spanning the week of the luteal phase (CD − 6 to CD 0) and 4 days spanning the week of the follicular phase (CD 4 to CD 10), with CD 0 set as the day before menstruation started. The EFA revealed a two-factor structure. Kaiser-Meyer-Olkin was 0.992, and Bartlett’s test of sphericity chi-square was 3653.89 (*P* < 0.001). However, the model fitness of CFA was found to be suboptimal (comparative fit index (CFI): 0.83, root mean square error of approximation (RMSEA): 0.12). Total scores for J-DRSP and the sum scores for each subscale were higher on CD 0 than on CD 10 (*p* < 0.001), suggesting validity for some criteria. ICC values for the total J-DRSP score from CD 0 to CD − 1, and between CD 9 to CD 10, were 0.60 (95% CI: 0.48–0.72) and 0.76 (95% CI: 0.69–0.82), respectively. Having eliminated some original items after considering factor loading for each item, we developed an 8-item Short-Form J-DRSP (J-DRSP (SF)) comprising 2 factors (S-Psychological and S-Physical, 4 items for each). CFA showed a better model fit (CFI: 0.99, RMSEA: 0.048), and ICC values in the luteal and follicular phases were 0.61 (95%CI: 0.51–0.68) and 0.70 (95%CI: 0.62–0.77), respectively.

**Conclusion:**

The J-DRSP has moderate to good reliability and a certain level of validity. The 8-item J-DRSP (SF) has a two-factor structure and can be used effectively among Japanese women to assess their PMS symptoms.

**Supplementary Information:**

The online version contains supplementary material available at 10.1186/s13030-021-00208-z.

## Background

Premenstrual syndrome (PMS) is the emotional and physical condition that appears in the luteal phase and resolves after menstruation starts. It is estimated that roughly 30% of women of reproductive age suffer from moderate to severe PMS [1]. If identified correctly, PMS can be controlled well and treated appropriately through lifestyle modifications and medication. Unfortunately, very few cases are identified or have their symptoms controlled, resulting in a large loss of productivity and major economic burden in Japan [2].

To accurately diagnose PMS, prospective daily recording for at least two consecutive menstrual cycles is critical, as it allows for the relationship between cycles and symptoms to be determined [3]. Of the available assessment tools, the Daily Record of Severity of Problems (DRSP) is the most widely used and is recommended by the Royal College of Obstetricians and Gynaecologists (RCOG) guidelines as a valid scale [3]. An algorithm for diagnosing Premenstrual Dysphoric Disorder (PMDD), named the Carolina Premenstrual Assessment Scoring System, based on the Diagnostic and Statistical Manual of Mental Disorders, fifth edition (DSM-V) using the DRSP has also been developed [4]. Although the DRSP was originally developed as a PMDD diagnostic tool, its use as a PMS scale has been reported in several studies [5, 6].

The Japanese version of the DRSP (J-DRSP) was developed in 2020 in accordance with the necessary translation and cultural adaptation guidelines published by the Patient-Reported Outcomes (PRO) Consortium [7, 8]. This process comprised two independent forward translations, two independent back translations, a consensus meeting about content equivalence and cultural adaptation, reviews by other native Japanese healthcare providers, and cognitive interviews with patients with PMDD at Kyoto University. Internal consistency and concurrent validity were confirmed for 119 Japanese women using the Center for Epidemiologic Studies Depression Scale, PMDD Scale, and the Numerical Rating Scale for general health [8].

The next necessary step in this process is to determine the factorial validity and test-retest reliability of the J-DRSP. In addition, development of a short-form of the J-DRSP would be ideal, as our previous study determined that the median time to complete the J-DRSP was 2 min, and both women with almost no premenstrual symptoms and those with very severe premenstrual symptoms complained that the use of the J-DRSP on a daily basis is cumbersome [8].

### Aim

This study aimed to assess the validity and reliability of J-DRSP, and to develop a short-form version of the J-DRSP.

## Methods

### Instruments

The original DRSP contains 21 items pertaining to premenstrual symptoms and 3 items to describe dysfunction in daily life caused by these symptoms, and is based on diagnostic criteria for PMDD from the DSM-IV (Table 1). All items are scored daily throughout the menstrual cycle on a scale of 1 (not at all) to 6 (extreme). The J-DRSP was developed according to the formal procedures outlined in the translation guidelines of the latest Patient-Reported Outcomes (PRO) Consortium [10].

**Table 1:** Original Daily Record of Severity of Problems [9]

Item	Symptoms
1	Felt depressed, sad, down, or blue
2	Felt hopeless
3	Felt worthless or guilty
4	Felt anxious, tense, keyed up, or on edge
5	Had mood swings (e.g., suddenly felt sad or tearful)
6	Was more sensitive to rejection or feelings were more easily hurt
7	Felt angry, irritable
8	Had conflicts or problems with people
9	Had less interest in usual activities (e.g., work, school, friends, hobbies)
10	Had difficulty concentrating
11	Felt lethargic, tired, fatigued, or had a lack of energy
12	Had increased appetite or overate
13	Had cravings for specific foods
14	Slept more, took naps, found it hard to get up when intended
15	Had trouble getting to sleep or staying asleep
16	Felt overwhelmed or that I could not cope
17	Felt out of control
18	Had breast tenderness
19	Had breast swelling, felt bloated, or had weight gain
20	Had headache
21	Had joint or muscle pain
22	At work, school, home, or in daily routine, at least one of the problems noted above caused reduced productivity or inefficiency
23	At least one of the problems noted above interfered with hobbies or social activities (e.g., avoided or did less)
24	At least one of the problems noted above interfered with relationships withothers
25	Menstrual bleeding

For the present study, we created the “DRSP-JAPAN” smartphone app, which allows users to log their daily symptoms for the J-DRSP. We released the app at the App Store and on Google Play for free in 2018 (Supplementary Fig. 1). Following user authentication, participants can rate their condition in the DRSP-JAPAN app and log their symptoms on the server every day. The app offers a visual display of daily condition, the severity of which is indicated by various colors and numbers, enabling participants to look back on the various changes in their symptoms over time.

The app has a blank check function, and a reminder mail is sent automatically if data have not been logged for more than three consecutive days.

### Participants

Participant recruitment flyers were posted between September 2018 and March 2019 at Kyoto University, Doshisha Women’s College of Liberal Arts, the staff lounge rooms of Kyoto University Hospital, and seven gynecology clinics in Japan where many asymptomatic women visit for medical check. Women were eligible for study participation if they were over 20 years of age, had self-reported regular menstruation, and used a smartphone or tablet. Those with any current mental disorder or who were taking oral contraceptives or ovulation inhibitors were excluded. All participants installed the DRSP-JAPAN app.

### Measurements

All data were collected through the DRSP-JAPAN app. At the beginning of the study, we collected demographic data on participant age and self-reported duration of menstruation. In addition to bleeding volume (one item of the J-DRSP), participants were prompted to log the start date of menstruation. Participant J-DRSP data are logged daily until the participants are notified that the research study has come to an end. Data from each cycle comprise those from half of the 7 days before menstruation starts and half of the days spanning day 4 to day 10 after menstruation begins. Once those data are collected, no further data are collected for that cycle.

### Statistical analysis

In order to assess factorial validity, we conducted exploratory factor analysis (EFA) using data for the 21 symptom items on the day before menstruation began. To measure the factorability of the correlation matrix, we conducted the Kaiser-Meyer-Olkin (KMO) test and Bartlett’s test of sphericity. Eigenvalues and screen plots were used to determine the number of factors, and promax rotation was used. Next, confirmatory factor analysis (CFA) was performed to assess the adoption. We then calculated the comparative fit index (CFI) and root mean square error of approximation (RMSEA) in order to analyze model fitness. CFI values range from 0 to 1, with larger values indicating a better fit. A CFI value of 0.95 or higher is regarded as a good fit [11]. RMSEA ranges from 0 to 1, with smaller values indicating a better model fit; values less than 0.06 indicate an acceptable model fit.

To confirm criterion validity, we compared the J-DRSP total score obtained the day before menstruation began to that obtained 10 days after the menstruation start date using the Wilcoxon signed-rank test. *P* < 0.05 was considered statistically significant.

To examine test-retest reliability, J-DRSP scores from each of the 2 days of the follicular phase and the luteal phase were compared. Specifically, with cycle day 1 (CD 1) and CD 0 set to represent the menstruation onset date and the day before the menstruation onset date, respectively, we compared data from CD 9 to CD 10 and CD − 1 to CD 0.

We determined the intraclass correlation coefficient (ICC), percent agreement, and weighted kappa coefficient. Percent agreement and kappa coefficients were calculated after the total J-DRSP was categorized into six levels. As a sensitivity analysis, ICC was determined for any participant whose symptoms interfered with daily life. Those were selected using J-DRSP items 22, 23, and 24, which concern dysfunction in productivity, social activities, and relationships by the symptom items from item 1 to 21, and those who scored 1 (none) for all of items 22, 23, and 24 were excluded. To measure internal consistency, we also calculated Cronbach’s alphas for the total J-DRSP score as well as for each subscale.

### Developing the Short-Form J-DRSP (J-DRSP (SF))

The short-form J-DRSP (J-DRSP (SF)) was created using classical test theory. First, items for which the factor load was less than 0.5 were excluded after conducting EFA for the original J-DRSP with promax rotation. Next, any items demonstrating sufficient model fit were explored using RMSEA and CFI. After developing the J-DRSP (SF), we conducted CFA and determined Cronbach’s alphas, ICCs, and criterion validity for the J-DRSP (SF) total score and each subscale. ICC values were calculated in both the follicular and luteal phases, i.e., CD 9 to CD 10 and CD-1 to CD 0, respectively. Sensitivity analysis was conducted again using J-DRSP items 22, 23, and 24. Stata 15.3 was used for all statistical analyses.

### Ethical considerations

The Kyoto University Ethics Committee approved the study protocol (R1593). Written informed consent was obtained from each participant.

## Results

### Participant demographics and characteristics

In total, 304 women (median age, 21 years; range, 20–50 years) gave their informed consent to participate in the study and successfully downloaded the app. There were 184 participants in their 20s, 67 in their 30s, 44 in their 40s, and 9 in their 50s.

The median number of recorded days was 33 (interquartile range, 22–47.5; range, 1–174). Of these, 243 logged their symptoms on at least 4 days of the week spanning the luteal phase (CD − 6 to CD 0) and 4 days of the week spanning the follicular phase (CD 4 to CD 10), with CD 1 set as the menstruation onset date. Data from these participants were analyzed.

### Validity and reliability of the J-DRSP

Data from the day before menstruation started (CD 0) were collected from 228 participants who logged symptoms on that day; these were used in the EFA. Eigenvalues and the scree plot suggested a two-factorial component, with a KMO of 0.992 and Bartlett’s test of sphericity chi-square of 3653.89 (*P* < 0.001), implying an adequate EFA with a sufficient sample size. Factor 1 and Factor 2 were labeled as the Psychological factor and Physical factor, respectively. The EFA coefficients for each item are shown in Table 2. The CFA revealed a CFI of 0.83 and RMSEA of 0.12.

**Table 2:** Exploratory Factor Analysis of the J-DRSP

	Factor 1	Factor 2	Uniqueness
3 felt worthless	**0.9951**	−0.1556	0.2038
5 mood swings	**0.8566**	0.0424	0.2133
1 depressed	**0.852**	0.0142	0.2569
2 hopeless	**0.8459**	−0.0083	0.2944
16 overwhelmed	**0.8139**	−0.0108	0.3498
4 tense	**0.788**	0.0951	0.2645
6 easily hurt	**0.7338**	0.1663	0.2619
10 difficulty concentrating	**0.5923**	0.2181	0.4197
8 conflict with people	**0.5137**	0.2898	0.4424
9 less interest	**0.4939**	0.3095	0.4449
17 out of control	**0.4885**	0.3619	0.3814
12 increased appetite	−0.1345	**0.7745**	0.5288
14 sleep more	0.0775	**0.6646**	0.4797
13 craving specific foods	−0.0397	**0.6433**	0.6206
11 lethargic	0.2059	**0.6431**	0.3575
19 weight gain	0.0781	**0.6196**	0.5419
7 irritable	0.33	**0.4594**	0.4665
15 trouble getting to sleep	0.1988	**0.4349**	0.6496
21 headache	0.1212	**0.395**	0.7618
20 joint or muscle pain	0.0638	**0.3311**	0.8566
18 breast tenderness	0.1062	**0.3185**	0.8397

Factor 1: Psychological Factor, Factor 2: Physcial Factor

Cronbach’s α of Factor 1 and Factor 2 were 0.95 and 0.84, respectively

Kaiser-Meyer-Olkin test: 0.922

Bartlett’s test of sphericity Chi-square: 3653.89 (*P* < 0.001)

**Fig. 1:**
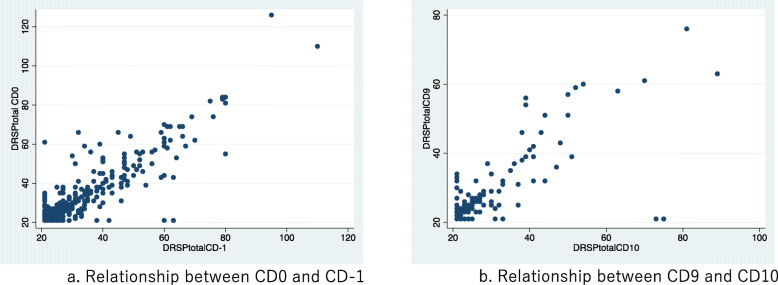
J-DRSP Test-Retest Reliability. **a**. Relationship between CD0 and CD-1. ICCs for the total J-DRSP score, Psychological factor, and Physical factor were 0.60 (0.50–0.68), 0.54 (0.44–0.63), and 0.58 (0.49–0.67), respectively. **b**. Relationship between CD9 and CD10. ICCs for the total J-DRSP score, Psychological factor, and Physical factor were 0.76 (0.69–0.82), 0.74 (0.66–0.80), and 0.72 (0.64–0.79), respectively. ICC: intraclass correlation coefficient. CD: cycle date, for which CD1 represents the menstruation start date

The total J-DRSP score was higher on CD 0 (median, 29) than on CD 10 (median, 24.5) (*p* < 0.001), and sum scores for each subscale were as well (*p* < 0.001 for all). ICCs for the total J-DRSP score for the period of CD 0 to CD − 1, and CD 9 to CD 10, were 0.60 (95% CI: 0.48–0.72) and 0.76 (95% CI: 0.69–0.82), respectively (Fig. 1). When all scores were categorized into 6 levels, percent agreement and weighted kappa values for the luteal phase and follicular phase were 98.9% and 0.76, and 97.9% and 0.57, respectively. A sensitivity analysis using the data set of symptomatic participants revealed that, when participants who scored 1 (none) for all of the items 22, 23, and 24 (interference of daily life due to the symptoms of items 1 to 21) on both CD − 1 and CD 0, or CD 9 and CD 10, were excluded, leaving 76 participants for the luteal phase and 30 for the follicular phase, ICCs between CD − 1 and CD 0 among the 76 participants, and between CD 9 and CD 10 among the 30 participants, were 0.79 (0.69–0.86) and 0.88 (0.68–0.86), respectively.

### Developing the J-DRSP (SF) and assessing its validity and reliability

**Fig. 2:**
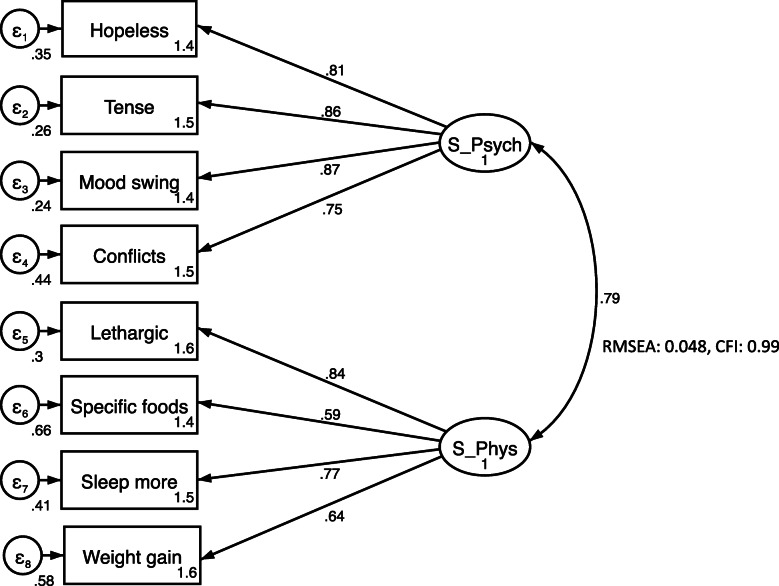
Confirmatory factor analysis results for J-DRSP(SF). Hopeless: Felt hopeless [Item 2]. Tense: Felt anxious, tense, keyed up, or on edge [Item 4]. Mood swing: Had mood swings (e.g., suddenly felt sad or tearful) [Item 5]. Conflicts: Had conflicts or problems with people [Item 8]. Lethargic: Felt lethargic, tired, fatigued, or had a lack of energy [Item 11]. Specific foods: Had cravings for specific foods [Item 13]. Sleep more: Slept more, took naps, found it hard to get up when intended [Item 14]. Weight gain: Had breast swelling, felt bloated, or had weight gain [Item 19]. S_Psych: Short-Form Psychological. S_Phys: Short-Form Physical

After items 7, 9, 15, 17,18, 20 and 21 were excluded due to low factor loading, J-DRSP (SF) was examined to ensure sufficient model fit. The final model was created and CFA was conducted (Fig. 2). Two factors were generated and labeled Short-Form Psychological (S-Psych) and Short-Form Physical (S-Phys). RMSEA was 0.048 and CFI was 0.99.

**Fig. 3:**
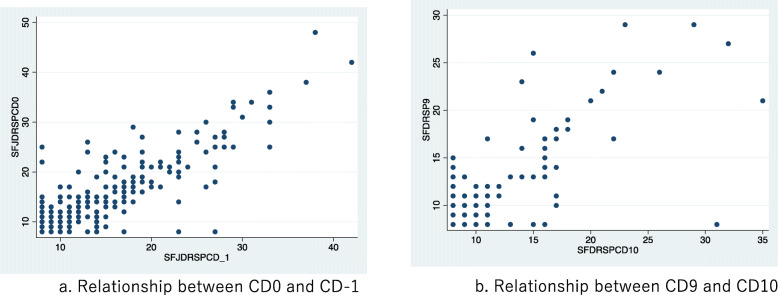
J-DRSP (SF) Test-Retest Reliability. **a**. Relationship between CD0 and CD-1. ICCs for the J-DRSP (SF) total score, SPsychological Factor, and S-Physical Factor were 0.61 (0.51–0.68), 0.52 (0.42–0.62), and 0.60 (0.50–0.68), respectively. **b** Relationship between CD9 and CD10. ICCs for the J-DRSP total score, SPsychological factor, and S-Physical factor were 0.70 (0.62–0.77), 0.68 (0.58–0.75), and 0.66 (0.56–0.74), respectively. ICC: intraclass correlation coefficient. CD: cycle date, for which CD1 represents the menstruation start date

Cronbach’s alphas for the J-DRSP (SF), S-Psych, and S-Phys were 0.89, 0.89, and 0.80, respectively. ICCs for the J-DRSP (SF), S-Psych, and S-Phys in the luteal and follicular phases were 0.61 (95%CI: 0.51–0.68) and 0.70 (95%CI: 0.62–0.77), respectively (Fig. 3). Sensitivity analysis revealed ICCs of 0.82 (0.73–0.88) for the J-DRSP (SF) in the luteal phase of 76 symptomatic women, and 0.84 (0.69–0.92) among 30 women. Scores for the J-DRSP (SF), S-Psych, and S-Phys on CD 0 were significantly higher than those obtained on CD 10 (*p* < 0.001).

## Discussion

In this study, daily J-DRSP records were collected from Japanese women using the DRSP-JAPAN smartphone app. According to the app’s function, there were no missing data when the participants recorded at least one item, the rate of DRSP completer was higher than the previous study and data management became easier [12]. Although CFA revealed suboptimal factorial validity of the J-DRSP, the newly developed J-DRSP (SF) showed an acceptable model fit. Test-retest reliability for the J-DRSP and J-DRSP (SF) were good, especially among those with significant PMS symptoms.

The original DRSP has 3 clinically determined subscales that comprise 6 items for Depressive Symptoms, 4 items for Physical Symptoms, and 2 items for Anger/Irritability [9]. There are 9 items left, and didn’t show the result of CFA. First, we conducted EFA, which indicated a two-factor structure. The CFA for a two-factor structure also revealed a suboptimal model fit, mainly because some items contributed to both factors. The total scores for J-DRSP were higher in the luteal phase, demonstrating some validity of the J-DRSP. Cronbach’s alpha was sufficiently high and test-retest reliability was good, especially among symptomatic women, indicating good reliability of the J-DRSP.

The newly developed J-DRSP (SF) contains 8 items in total (4 psychological and 4 physical items). The CFA of the J-DRSP (SF) showed a better model fit than that of the original J-DRSP, suggesting higher validity as a scale to assess PMS symptoms among Japanese women. ICC and Cronbach’s alpha also indicated sufficient reliability of the J-DRSP (SF).

In clinical settings, J-DRSP (SF) in the luteal phase could be used as a self-awareness checklist for women who may not yet realize that their symptoms are related to PMS. In addition, checking the J-DRSP (SF) could help identify whether their main problems are more physical or psychological in nature, which could help with effective clinical management of PMS.

This study has some limitations. First, none of our participants were patients, and those with and without subjective PMS symptoms were included in the analysis. The original DRSP was developed as a PMDD diagnostic tool and validated as a PMDD scale, although it is widely used for PMS. Floor effects can explain the lower ICC in this study relative to results from the original DRSP [9]. Second, since most participants interacted with the researchers through the internet, the ability to verify their identity and the accuracy of symptom records was limited. Third, more than half of the study participants were in their 20s, and the sample size might be insufficient to ensure a stable factorial analysis, which limits generalizability.

Finally, diagnostic properties of the J-DRSP (SF) were not analyzed in this study. The original DRSP is used as a diagnostic tool to clarify the relationship between PMS-like symptoms and the menstrual cycle. Future studies should compare J-DRSP (SF) to the DRSP as a diagnostic tool.

## Conclusion

The J-DRSP exhibited moderate to good reliability and certain level of validity. The 8-item J-DRSP (SF), with its two-factorial structure, can be used as an optimized form of the J-DRSP to evaluate PMS symptoms among Japanese women.

## Supplementary Information

**Additional file 1: Supplementary Figure 1.** Screenshots of the DRSP-JAPAN app.

## Data Availability

The datasets generated and analyzed during the current study are not publicly available, as we did not have participant consent for the secondary use of their data.

## Abbreviations

CD: Cycle day
CFA: Confirmatory factor analysis
CFI: Comparative fit index
DSM: Diagnostic and Statistical Manual of Mental Disorders
EFA: Exploratory factor analysis
ICC: Intraclass correlation
J-DRSP: Japanese Version of the Daily Record of Severity of Problems
J-DRSP (SF): Short-Form Japanese Version of the Daily Record of Severity of Problems
KMO: Kaiser-Meyer-Olkin
PMDD: Premenstrual Dysphoric Disorder
PMS: Premenstrual syndrome
PRO: Patient-Reported Outcomes
RCOG: Royal College of Obstetricians and Gynaecologists
RMSEA: Root mean square error of approximation
