# Correction to: The reliability and validity of the Japanese version of the daily record of severity of problems (J-DRSP) and development of a short-form version (J-DRSP (SF)) to assess symptoms of premenstrual syndrome among Japanese women

**DOI:** 10.1186/s13030-021-00231-0

**Published:** 2021-12-31

**Authors:** Yumie Ikeda, Miho Egawa, Kazuya Okamoto, Masaki Mandai, Yoshimitsu Takahashi, Takeo Nakayama

**Affiliations:** 1 Department of Health Informatics, Kyoto University School of Public Health, Konoecho Yoshida, Sakyo-ku, Kyoto City, 606-8303 Japan; 2 Department of Obstetrics and Gynecology, Kyoto University Hospital, 54 Kawaramachi Shogoin, Sakyo-ku, Kyoto City, 606-8397 Japan; 3 Department of Medical Informatics, Kyoto University Hospital, 54 Kawaramachi Shogoin, Sakyo-ku, Kyoto City, 606-8397 Japan

**Correction to:** ***BioPsychoSocial Med*** **15, 6 (2021).**

10.1186/s13030-021-00208-z

Following publication of the original article [1], the authors reported the additional information, which are marked in red in the attached Word file (Additional file 1).

(1) In the Methods section (Line 4 ~ 6, Page 9), the below sentences are added:

In order to confirm the criteria validity for J-DRSP (SF), we calculated Pearson’s coefficients, with *p* values, between the total score of the 8 items of J-DRSP (SF) and J-DRSP.

Two sided tests were done with a significance level of 0.001.

(2) In the Results section (Line 15 ~ 16, Page 11), the below sentence is added:

The result of correlation coefficient between J-DRSP(SF) and J-DRSP was 0.97, with p value <0.0001.

The original article [1] has been corrected.

## Supplementary Information

**Additional file 1.** Revised manuscript.
