# Supplementary figures and images for: The reliability and validity of the Japanese version of the Daily Record of Severity of Problems (J-DRSP) and Development of a Short-Form version (J-DRSP (SF)) to assess symptoms of premenstrual syndrome among Japanese women
Source: Biopsychosoc Med. 2021 Mar 18;15:6. doi: 10.1186/s13030-021-00208-z (PMC7977312; doi:10.1186/s13030-021-00208-z)

## Slide 1
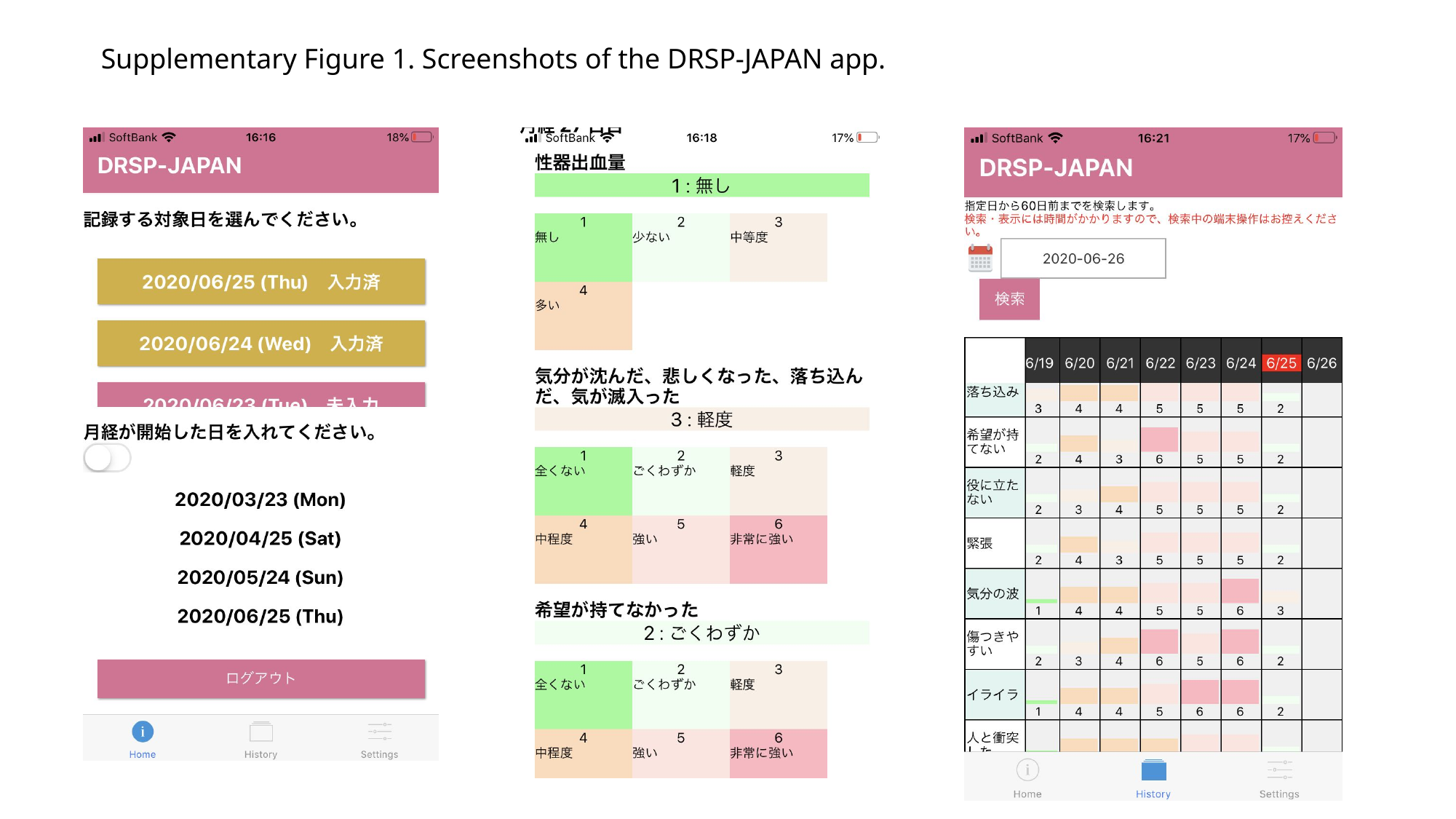

Supplementary Figure 1. Screenshots of the DRSP-JAPAN app.

Supplement: Supplementary file 1 — Additional file 1: Supplementary Figure 1. Screenshots of the DRSP-JAPAN app. [file 13030_2021_208_MOESM1_ESM.pptx]
